# The Effectiveness of Herbal Mixture of *Echium amoenum* L. and *Rheum ribes* on Obsessive‐Compulsive Disorder: A Randomized Controlled Triple‐Blind Clinical Trial

**DOI:** 10.1002/npr2.70073

**Published:** 2025-11-17

**Authors:** Ali Talaei, Ala Masiha, Fahimeh Afzaljavan, Mohammad Reza Noras, Hasan Rakhshandeh, Lida Jarahi, Kiana Sedighi

**Affiliations:** ^1^ Psychiatry and Behavioral Sciences Research Center Mashhad University of Medical Sciences Mashhad Iran; ^2^ Department of Persian Medicine, School of Complementary and Persian Medicine Mashhad University of Medical Sciences Mashhad Iran; ^3^ Pharmacological Research Center of Medicinal Plants Mashhad University of Medical Sciences Mashhad Iran; ^4^ Department of Community Medicine, Faculty of Medicine Mashhad University of Medical Sciences Mashhad Iran

**Keywords:** *Echium amoenum* L., obsessive‐compulsive disorder, *Rheum ribes*, Yale‐Brown Scale

## Abstract

**Objective:**

The use of complementary and alternative medicine has gained traction in the treatment of psychiatric disorders, including obsessive‐compulsive disorder (OCD). This trial investigates the efficacy of an herbal combination of *Echium amoenum* L. and *Rheum ribes* in reducing OCD symptoms.

**Methods:**

In this triple‐blind, randomized, controlled clinical trial, 40 participants were assigned to either the intervention group, receiving a daily dose of 30 mL of the herbal syrup, or a control group receiving a placebo. OCD severity was measured using the Yale‐Brown Obsessive Compulsive Scale (Y‐BOCS) at baseline, and at 4, 6, and 8 weeks. Side effects were monitored based on a preestablished checklist. Data were analyzed using SPSS v 16.

**Results:**

There was no significant difference between groups in age (*p* = 0.214) and sex (*p* = 1.00). The change in OCD scores between times significantly differed in the intervention group (*p* = 0.037). However, the OCD score did not change significantly during the time in the control group (*p* = 0.932). Moreover, there was no significant difference between the two groups at each time. However, the trend of changes during the trial was significant between the groups (*p* < 0.001). Additionally, no adverse effects were reported among participants consuming the herbal syrup.

**Conclusion:**

This study provides evidence that *Echium amoenum* L. and *Rheum ribes* can effectively decrease OCD severity without adverse effects, suggesting their potential as a safe treatment option. Future research with larger sample sizes and longer follow‐up periods is warranted to confirm these findings and explore the mechanisms of action.

**Trial Registration:**

Iranian Registry for Clinical Trials (registration number: IRCT20200712048088N1)

## Abstract 2

A randomized controlled trial demonstrates that a daily dose of herbal syrup combining *Echium amoenum* L*.* and *Rheum ribes* significantly reduces obsessive‐compulsive disorder (OCD) symptoms without adverse effects. This safe, plant‐based treatment offers a promising adjunct to conventional OCD therapies.

## Graphical abstract

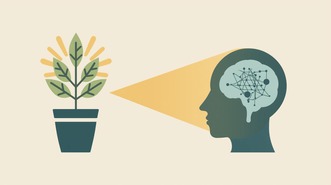

## 1 Introduction

Obsessive‐compulsive disorder (OCD) is a prevalent mental health condition characterized by intrusive thoughts, images, and urges, accompanied by compulsive behavior or mental acts designed to alleviate anxiety [1, 2]. Symptoms of OCD can be classified into three categories, including obsessive thoughts, compulsive behaviors, or a combination of both [3]. Despite recognizing the excessive nature of these thoughts and behaviors, individuals with OCD often struggle to control or resist them [4]. While compulsive actions aim to mitigate anxiety stemming from obsessions [5], relief is typically short‐lived, and anxiety levels may rebound or escalate in subsequent days [6].

Recent studies indicate that the prevalence of OCD in the general population ranges from 2% to 3%, with as many as 10% of individuals seeking treatment for psychiatric disorders affected by it [7, 8]. Gender differences in prevalence exist: among adults, OCD is slightly more common in females, while it is more frequently diagnosed in males during adolescence [9]. Individuals with OCD often experience comorbid conditions, including generalized anxiety disorder, specific phobias, substance misuse, panic disorder, eating disorders, and personality disorders.

The treatment of anxiety disorders, including OCD, is a fundamental aspect of psychiatric practice [10, 11]. Current evidence‐based treatments include serotonin reuptake inhibitors, tricyclic medications such as clomipramine, and cognitive‐behavioral therapy (CBT), particularly exposure and response prevention (ERP) [12]. Despite significant advancements in managing OCD, some patients exhibit limited therapeutic responses, and many experience difficulty tolerating the distress associated with ERP [13]. This has led to increased interest in traditional complementary and alternative medicine approaches [14].

Several plant‐based therapies have been explored for OCD management, including *Hypericum perforatum* [15], *Silybum marianum* (*Mehdi* [16]), *Curcumin* [17], *Echium amoenum L* (Borage) [18], and *Rheum ribes* [15, 19]. Borage, a traditional Persian herb, is recognized for its anxiolytic, sedative, and antidepressant properties, demonstrating efficacy in OCD treatment [18]. Similarly, *R. ribes* , commonly known as rhubarb, has been associated with reduced symptoms of obsession and compulsion in OCD patients [20]. While preliminary studies suggest that these herbal interventions may hold promise, there is a pressing need for further double‐blind, randomized controlled trials employing rigorous methodologies to assess the effectiveness of these alternative treatments.

This study aimed to evaluate the impact of a combination of *Echium amoenum* L and *Rheum ribes* extracts on the severity of OCD symptoms in affected individuals.

## 2 Methods and Materials

### 2.1 Ethical

The study received approval from the Research Ethics Committee of Mashhad University of Medical Sciences (Ethical approval number: IR.MUMS.MEDICAL.REC.1399.391). Additionally, the study protocol was registered with the Iranian Registry for Clinical Trials (registration number: IRCT20200712048088N1). All study procedures were conducted in accordance with the principles of medical ethics as established by the Ministry of Health and Medicine, the Declaration of Helsinki, and the Medical Ethics Committee of Mashhad University of Medical Sciences. Participant information was anonymized using unique codes to safeguard confidentiality. Informed consent was obtained from all participants, who were also assured of their right to withdraw from the study at any time without any repercussions.

### 2.2 Study Design

This study was a triple‐blind, randomized, controlled clinical trial conducted at Ibn‐E‐Sina Psychiatric Hospital, Mashhad University of Medical Sciences, Mashhad, Iran, in 2020.

### 2.3 Sample Size Calculation

As this was a pilot study and no prior clinical trials had evaluated the combined effectiveness of *Echium amoenum* L. and *Rheum ribes* for OCD, a formal power calculation was not performed. Instead, a sample size of 40 patients (20 per group) was selected based on standard recommendations for pilot RCTs in psychiatric research, which suggest 30–50 participants to assess feasibility, estimate effect sizes for future trials, and detect preliminary signals of efficacy [21]. This size allowed for the detection of moderate effect sizes with reasonable precision while accounting for potential attrition.

### 2.4 Study Population

Patients diagnosed with OCD who were referred to the outpatient clinic of Ibn‐E‐Sina Hospital in 2020 were recruited. The diagnosis was established based on the DSM‐5 criteria, and participants with mild to moderate OCD severity, as determined by the Yale‐Brown Obsessive Compulsive Scale (Y‐BOCS), were included in the study.

### 2.5 Inclusion and Exclusion Criteria

Inclusion criteria consisted of a definitive diagnosis of OCD according to DSM‐5, a Y‐BOCS score of 10 or higher, educational attainment beyond a high school diploma, age between 18 and 50, and no use of chemical medications within the past 8 weeks. Participants were required to have completed at least 20 h of exposure and response prevention therapy (either individually or in groups) and to be unwilling to use pharmacological treatments.

Exclusion criteria included the presence of psychotic symptoms or bipolar disorder, as well as individuals initiating new psychotherapeutic or psychiatric treatments during the study period.

### 2.6 Randomization and Blinding

Participants were randomly assigned to either the intervention or placebo group using a computer‐generated random number table for sequence generation. Allocation concealment was achieved through sequentially numbered, sealed opaque envelopes prepared by an independent third party. This study employed a triple‐blind design, ensuring that the assignment was concealed from participants, experimenters, and researchers analyzing the data. Blinding was maintained by having the pharmacist prepare identical‐looking syrups for both groups, matched in taste (sweetened with simple syrup), color (using food coloring), and viscosity (adjusted via alcohol‐water ratio). The blinding code was broken only after data analysis was complete.

### 2.7 Intervention

To prepare the aqueous‐alcoholic extract, the petals of *Echium amoenum* L. and the roots of *Rheum ribes* were utilized. Plant materials were sourced from a certified local herbal supplier in Mashhad, Iran, and authenticated morphologically by a qualified botanist at the Herbarium of Mashhad University of Medical Sciences based on standard taxonomic references. Specifically, 1 cc of *Echium amoenum* extract (17 mg dry weight equivalent) and 1 cc of *Rheum ribes* extract (15 mg dry weight equivalent) were dissolved in 30 mL of water and 70 mL of alcohol, with simple syrup added to create the final syrup formulation. Extracts were prepared using a standard aqueous‐alcoholic maceration process (1:5 plant‐to‐solvent ratio, 70% ethanol, 48‐h maceration at room temperature, followed by filtration and concentration under reduced pressure). No formal standardization to specific phytochemical markers (e.g., rosmarinic acid for *E. amoenum* or anthraquinones for *R. ribes* ) was conducted; dosing was standardized based on dry weight equivalents to ensure consistency. The placebo consisted of 660 mg of sugar and a food coloring solution mixed in 1 L of water. A pharmacist prepared the placebo to match the flavor and packaging of the intervention.

To ensure reproducibility and minimize degradation, the syrup was freshly prepared weekly and stored in opaque glass bottles at 4°C (refrigerated) until distribution to participants. Shelf‐life stability was not formally assessed; however, no signs of spoilage (e.g., changes in color, odor, or precipitation) were observed during the 8‐week study period. Participants in the intervention group received 30 mL of the herbal mixture daily, administered as 10 mL before each meal (morning, noon, and evening). The control group received the placebo concurrently with the intervention subjects.

### 2.8 Outcome Measurements and Instrument

The primary outcome measure was the severity of OCD, assessed using the Yale‐Brown Obsessive Compulsive Scale (Y‐BOCS) at baseline (week 0), and at weeks 4, 6, and 8. The Y‐BOCS is a semi‐structured interview designed to evaluate the intensity of obsessions and compulsions, independent of their number and content. Unlike other assessment tools, the Y‐BOCS is known for its high sensitivity to therapeutic changes and is widely utilized to evaluate the efficacy of both pharmacological and psychological treatments for OCD. It is a clinician‐rated, 10‐item scale, with each item scored from 0 (no symptoms) to 4 (extreme symptoms), yielding a total score range of 0–40, and separate subtotals for the severity of obsessions and compulsions [22].

### 2.9 Side Effect Consideration

In line with prior studies, a preestablished checklist was employed to evaluate the adverse effects associated with the prescribed treatments. This study specifically monitored six potential side effects: nausea, headache, blurry vision, dry mouth, constipation, and drowsiness.

### 2.10 Adherence Assessment

Adherence to the 30 mL daily dose was monitored through participant self‐reported daily logs and by measuring returned syrup bottles at each follow‐up visit (weeks 4, 6, and 8). Nonadherence was defined as missing > 20% of doses; no participants met this criterion.

### 2.11 Statistical Analysis

Data analysis was conducted using SPSS software. Descriptive statistics, including measures of central tendency, dispersion indices, and frequency distributions, were calculated to characterize the study population. Chi‐square tests were employed to compare qualitative variables between study groups. For quantitative variables, normality was assessed using Shapiro–Wilk tests, homogeneity of variances using Levene's tests, and sphericity using Mauchly's test. Either independent *t*‐tests or Mann–Whitney *U* tests were utilized for comparisons between the treatment and control groups. Additionally, mixed repeated measures ANOVA was conducted to analyze trends over time between groups, with Greenhouse–Geisser corrections applied if sphericity was violated. Post hoc pairwise comparisons were performed using paired *t*‐tests (within groups) and independent *t*‐tests (between groups), with Bonferroni corrections for multiple comparisons. Effect sizes (partial *η* ^2^ for ANOVA effects, Cohen's *d* for group differences) and 95% confidence intervals for means were calculated. A significance level of *p* < 0.05 was established for all statistical tests.

## 3 Results

**FIGURE 1:**
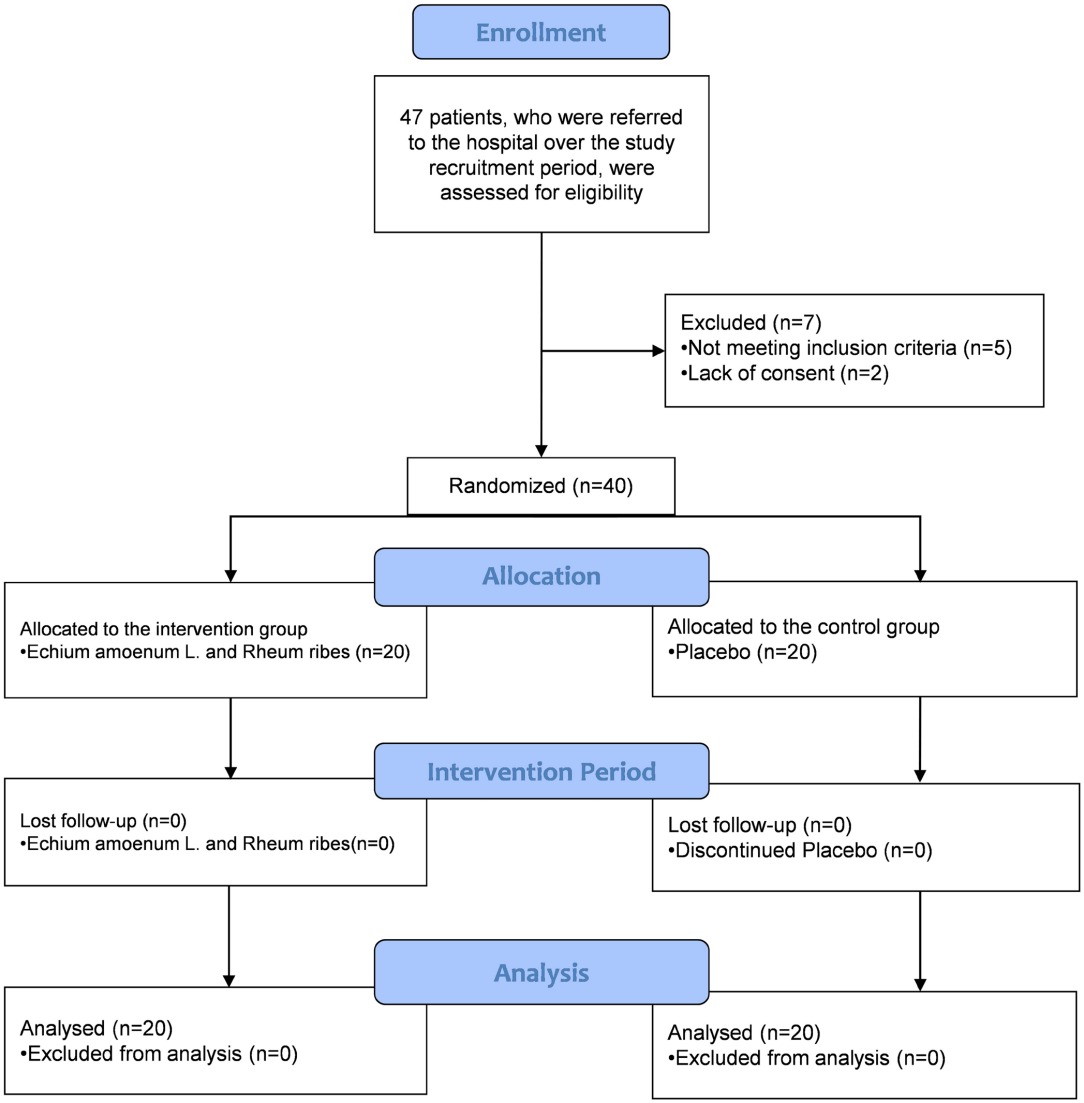
CONSORT flow diagram illustrating the study population, including enrolment, allocation, follow‐up, and analysis of participants in the randomized controlled trial.

The procedure of study based on the CONSORT diagram is shown in Figure 1. Forty‐seven OCD patients who were referred to the Ebn‐e‐Sina hospital over the study recruitment period in 2020, were assessed for eligibility. Seven subjects were excluded due to the lack of consent (*n* = 2), comorbidities (*n* = 4), and pregnancy (*n* = 1).

The characteristics of the study population are summarized in Table 1. The mean ages were 31.3 ± 11.1 years in the control group and 35.2 ± 10.4 years in the intervention group. There were no significant differences between the groups in terms of age (*p* = 0.214) or sex (*p* = 1.00).

**TABLE 1:** Baseline characteristics of the study population, including age (mean ± SD and categorical distribution) and sex distribution, with *p* values for between‐group comparisons.

Characteristics	Group	Control	Intervention	*p*
Age (Years)	Mean	31.3 ± 11.1	35.2 ± 10.4	0.214
	≤ 18	0 (0%)	1 (5%)	0.455
	19–30	10 (50%)	8 (40%)	
	31–50	10 (50%)	11 (55%)	
Sex	Male	4 (20%)	5 (25%)	1.00
	Female	16 (80%)	15 (75%)	

**FIGURE 2:**
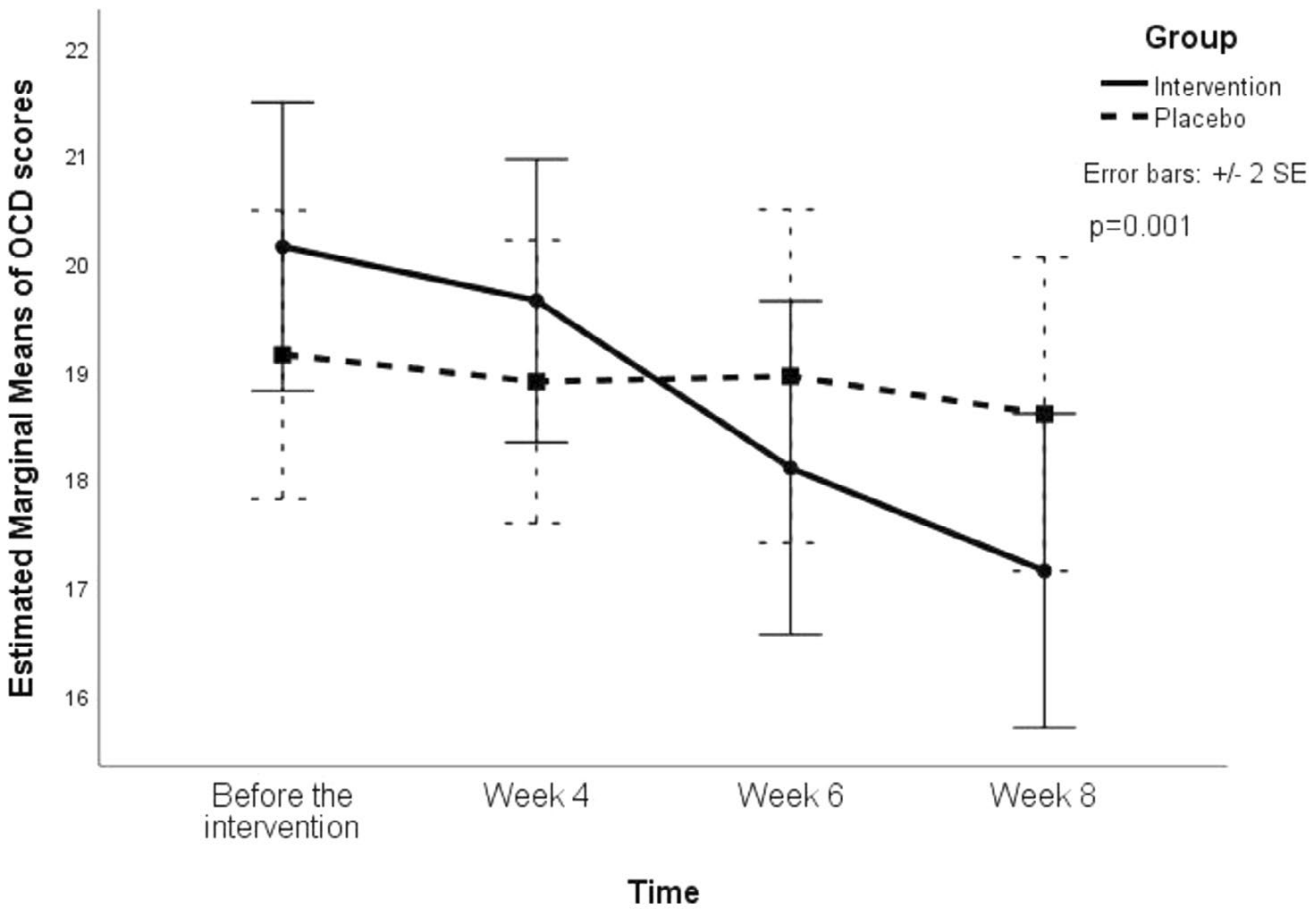
Trend of changes in OCD scores (measured by Y‐BOCS) over different weeks in the control and intervention groups, showing the mean scores at baseline, week 4, week 6, and week 8.

Table 2 presents the OCD scores for both the control and intervention groups at various time points. Normality was assessed using Shapiro–Wilk tests for each group‐time combination; the data were normally distributed in 7 out of 8 combinations (all *p* > 0.05), with a minor violation in the control group at week 6 (*p* = 0.045). Given the robustness of ANOVA to minor normality violations and our sample size (*n* = 20 per group), no transformations were applied. Homogeneity of variances between groups was confirmed at each time point using Levene's tests (all *p* > 0.05). The assumption of sphericity was violated for the overall model (Mauchly's *W* = 0.003, *p* < 0.001), as well as for separate within‐group analyses (intervention: Mauchly's *W* = 0.004, *p* < 0.001; control: Mauchly's *W* = 0.001, *p* < 0.001). Therefore, Greenhouse–Geisser corrections were applied to the degrees of freedom for the time and group × time effects (overall *ε* = 0.37; intervention *ε* = 0.40; control *ε* = 0.27). The change in OCD scores over time was statistically significant in the intervention group (*F*(1.19, 22.66) = 8.50, *p* = 0.006, partial *η* ^2^ = 0.309; Greenhouse–Geisser corrected, *ε* = 0.40), but not in the control group (*F*(0.81, 15.35) = 2.96, *p* = 0.110, partial *η* ^2^ = 0.135; Greenhouse–Geisser corrected, *ε* = 0.27). Additionally, there were no significant differences in OCD scores between the two groups at the specified time points. As illustrated in Table 3 and Figure 2, the trend of changes throughout the trial was significantly different between the groups (*F*(1.11, 42.18) = 5.91, *p* = 0.017, partial *η* ^2^ = 0.135; Greenhouse–Geisser corrected, *ε* = 0.37). There was no significant main effect of group (*F*(1, 38) = 0.02, *p* = 0.880, partial *η* ^2^ < 0.001). Cohen's *d* for group differences (intervention minus control) at each time point were as follows: baseline: *d* = 0.33; week 4: *d* = 0.26; week 6: *d* = −0.25; week 8: *d* = −0.45.

**TABLE 2:** Comparison of OCD scores (mean [95% CI] ± SD) between the intervention and control groups at baseline, week 4, week 6, and week 8, with within‐group *p* values for change over time and between‐group *p* values at each time point.

Group	Before	Week 4	Week 6	Week 8	*p* ^a^
Control	19.15 [17.89, 20.41] ± 2.7	18.90 [17.64, 20.16] ± 2.69	18.95 [17.77, 20.13] ± 2.52	18.60 [17.32, 19.88] ± 2.74	0.110
Intervention	20.15 [18.63, 21.67] ± 3.25	19.65 [18.18, 21.12] ± 3.15	18.10 [16.14, 20.06] ± 4.18	17.15 [15.42, 18.88] ± 3.67	**0.006**
*p* ^a^	0.296	0.423	0.441	0.166	

^a^ *p* values for the time effect within each group are based on separate repeated measures ANOVAs with Greenhouse–Geisser corrections applied due to sphericity violations [intervention: *ε* = 0.40; control: *ε* = 0.27]. Between‐group *p* values are unadjusted from independent *t*‐tests at each time point, as no significant differences were found even before multiple comparison corrections. Significant *p* values are shown in bold.

**TABLE 3:** Results of repeated measures ANOVA comparing the change trend of OCD scores between the intervention and control groups during the study.

Source	Type III sum of squares	Adjusted df^a^	Mean square	*F*	Adjusted *p* value^a^	Partial eta squared
Time	75.019	1.11	25.006	10.287	0.002	0.213
Time × Group	43.119	1.11	14.373	5.913	0.017	0.135

^a^ Degrees of freedom and *p* values adjusted using Greenhouse–Geisser correction due to sphericity violation [*ε* = 0.37].

Post hoc pairwise comparisons (paired *t*‐tests within groups and independent *t*‐tests between groups) with Bonferroni correction revealed significant reductions in OCD scores in the intervention group from baseline to week 8 (adjusted *p* = 0.022) and from week 6 to week 8 (adjusted *p* = 0.007). No significant changes were found in the control group or between groups at any time point after correction.

Based on a checklist of known side effects derived from relevant literature and traditional Iranian medicine sources, no adverse effects were reported in patients receiving treatment.

## 4 Discussion

In the past decade, complementary and alternative medicine has gained popularity as a treatment approach for various diseases, particularly psychiatric disorders. Several supplements and herbal medicines have been explored for their efficacy in treating obsessive‐compulsive disorder (OCD), with a focus on plant‐based treatments such as *Echium amoenum* L. and *Rheum ribes*. Observations suggest these herbs may possess anti‐OCD properties. The present clinical trial evaluated the effectiveness of a combination of *Echium amoenum* L. and *Rheum ribes* on OCD severity. Our findings indicated that a daily dose of 30 mL of the herbal syrup significantly improved OCD symptoms. Importantly, no adverse effects were reported following the consumption of the *Echium amoenum* L.–*Rheum ribes* syrup throughout the study.

Results demonstrated a significant reduction in Y‐BOCS scores in the intervention group compared to the control group during the study period. A previous study involving a daily dose of 500 mg of *Echium amoenum* extract [18] in a 6‐week double‐blind, parallel‐group trial found no significant differences in the trend of OCD score changes. However, that study did report significant efficacy of the *Echium amoenum* aqueous extract compared to placebo in reducing anxiety and obsessive‐compulsive symptoms at weeks 4 and 6, with no notable differences in adverse effects between groups.

Another study examined the potential benefits of a syrup combining *Echium amoenum* and *Melissa officinalis* alongside fluvoxamine over 8 weeks. Consistent with our findings, the treatment group showed a significant reduction in Y‐BOCS scores by the eighth week; however, there was no significant difference in the trend of OCD score changes between groups during the trial [23]. Additionally, research on a hydroethanolic extract of *Rheum ribes* indicated a reduction in OCD symptoms with a daily dose of 1200 mg [24]. Although the efficacy of these herbs remains a topic of debate, these findings underscore the potential anti‐OCD effects of *Echium amoenum* and *Rheum ribes*. Variability in study outcomes may be attributed to factors such as dosage, duration of consumption, specific herbal compounds, and the severity of the disorder.

The significant interaction between group and time, despite no significant between‐group differences at individual time points, suggests a cumulative treatment effect over time. Clinically, this indicates that the herbal mixture may lead to gradual symptom improvement, potentially meaningful for patients with mild‐to‐moderate OCD who prefer non‐pharmacological options, though the lack of point‐wise differences highlights the need for longer trials to confirm sustained benefits.

This study is the first clinical trial investigating the efficacy of a combined mixture of *Echium amoenum* L. and *Rheum ribes* on OCD severity. However, several limitations should be acknowledged. The simultaneous effects of selective serotonin reuptake inhibitors (SSRIs) and the herbal mixture were not assessed in both treatment and control groups. Furthermore, the potential beneficial effects of this herbal combination on patients' digestion, nervous system, sleep patterns, or appetite were not evaluated. Since a fixed syrup dose was administered, we could not determine the therapeutic and toxic effect thresholds. Additionally, the study did not analyze gender‐based treatment effects due to the low number of male participants. Laboratory testing was not conducted to assess side effects, relying solely on a checklist, which may miss subclinical changes. Additionally, the study did not perform formal plant material standardization to specific phytochemical markers or shelf‐life stability testing, which could affect reproducibility in larger replications. Future studies should incorporate these analyses (e.g., HPLC quantification of rosmarinic acid and anthraquinones) to better characterize the extracts. Laboratory testing was not conducted to assess side effects. Other limitations include the short duration (8 weeks) without posttreatment follow‐up, potentially underestimating long‐term effects or relapse, and the absence of mechanistic exploration (e.g., testing hypothesized serotonin modulation via biomarkers).

To further elucidate the effects of these herbal extracts on OCD, expanding the sample size, conducting long‐term studies, examining the impact of the herbal extract in conjunction with SSRI medications, and exploring dose–response relationships could be recommended.

## 5 Conclusion

In conclusion, this clinical trial demonstrates that the combination of *Echium amoenum* L. and *Rheum ribes* may significantly reduce the severity of OCD symptoms, as evidenced by the marked improvement in Y‐BOCS scores in the treatment group compared to the control group, without any reported adverse effects based on monitoring. These findings suggest that this herbal syrup may offer a safe adjunctive treatment for OCD, addressing a critical need in psychiatric care. However, further studies with larger sample sizes, longer durations, post‐treatment follow‐ups, and controlled designs are necessary to explore the long‐term efficacy, optimal dosing, mechanistic pathways (e.g., serotonin modulation), and potential interactions with conventional therapies such as SSRIs. Ultimately, this research expands the understanding of complementary and alternative approaches in managing OCD, reinforcing the potential of herbal medicines in psychiatric treatment while highlighting the need for cautious interpretation in pilot contexts.

## Author Contributions

Conceptualization: A.T., M.R.N., and A.M.; Methodology: A.T., M.R.N., A.M., H.R., L.J., K.S., and F.A.; Formal analysis and investigation: F.A., A.T., and L.J.; Writing – original draft preparation: K.S., A.T., and F.A.; Writing – review and editing: K.S., A.T., and F.A.; Funding acquisition: A.T.; Supervision: A.T., M.R.N., and F.A. All authors checked and approved the final version of the manuscript for publication in the present journal.

## Ethics Statement

Approval of the Research Protocol by an Institutional Reviewer Board: This study was presented to the Ethics Committee of the Medical School and registered under the code “IR.MUMS.MEDICAL.REC.1399.391”.

## Consent

The authors have nothing to report.

## Conflicts of Interest

The authors declare no conflicts of interest.

## Supporting information


**Appendix S1:** Supporting Information.

## Data Availability

The datasets generated and analyzed during the current study are available in the Appendix S1.
